# New gene sensors enable precise cell monitoring and control without altering gene sequence

**DOI:** 10.1093/synbio/ysae014

**Published:** 2024-10-29

**Authors:** Tea Crnković

**Affiliations:** Department of Biological Engineering, Massachusetts Institute of Technology, Cambridge, MA 02139, USA

From glucose meters used by diabetics to monitor blood sugar levels to home pregnancy tests, biosensors are widely used in everyday life to detect biological changes and provide critical diagnostic information [1]. Biosensors are tools that can detect biomolecules, pathogens, or changes in a cell’s internal state, such as fluctuations in gene expression and these sensors are essential for diagnostic applications like tracking disease progression, or even ensuring quality control in biomanufacturing processes. While conventional methods such as CRISPR-Cas9 [2] or RNA-based sensors [3, 4] allow for the interrogation of gene expression, they involve genetic modifications that could potentially alter how the gene operates within the cell. This issue has been particularly problematic in the development of therapeutic applications, where maintaining the integrity of natural gene function is essential. To address these issues, researchers from MIT led by Prof. Ron Weiss and Prof. Jim Collins have developed a gene-sensing technology that allows monitoring of gene activity without altering its genetic code, while also triggering a cellular response based on that activity [5]. This approach can be used to develop targeted treatments for conditions like cellular stress-related diseases, including neurodegenerative disorders and diabetes, by conditionally activating protective proteins to mitigate cell damage.

The MIT researchers integrated genetic biosensors into cells by inserting synthetic guide RNAs (sgRNAs) into the terminator region of a gene, a location that does not interfere with the gene’s natural coding sequence. Once the sgRNAs are produced, they can bind to synthetic activators, triggering a downstream response, such as the expression of a fluorescent protein that signals changes in the cell, or a therapeutic protein. By varying the number of sgRNA cassettes integrated into the terminator region, researchers can control the level of response triggered by gene expression. This dosage control enables fine adjustments in how cells react to changes in gene activity, a useful tool to study gene regulation in a highly controlled environment.

The MIT team also developed a key component of their gene-sensing platform called SynPAS, a synthetic polyadenylation sequence designed to ensure efficient sgRNA generation without disrupting natural RNA processing and gene expression. SynPAS insulates the upstream gene from the activity of the sgRNA cassette inserted in the terminator region. This development is essential to prevent interference with the normal transcription and translation of genes within genome where surrounding functional elements are not fully known. This allows the sensor to monitor gene activity without affecting the cell’s normal functions, making it a robust tool for studying gene regulation in complex biological systems.

Next, the MIT researchers demonstrated the potential of their gene-sensing platform through chromosomal integration into the *RPS21* gene, which is constitutively expressed in HEK293FT cells. By embedding the gene sensor module into the *RPS21* locus, they were able to monitor real-time gene expression without modifying the gene’s coding sequence. The integrated gene sensor successfully generated functional sgRNAs, which activated a downstream fluorescent reporter. This experiment demonstrated the viability of using the gene sensor for long-term monitoring of endogenous gene activity within its natural chromosomal context.

Lastly, the researchers demonstrated one practical application of the gene-sensing platform by integrating the sensor into the *Herpud1* gene, which is involved in the unfolded protein response (UPR) pathway. This pathway is activated when proteins in the cell become misfolded, triggering stress responses that help restore cellular balance. The researchers inserted sgRNA cassettes into the terminator region of *Herpud1* in CHO-K1 cells, which were then exposed to endoplasmic reticulum stress. The gene sensor detected the activation of *Herpud1*, which activated downstream production of the anti-apoptotic protein BCL-2, which helps cells survive under stress. By adjusting the number of sgRNA cassettes, the researchers could fine-tune the strength of the response, with cells producing more BCL-2 when more sgRNAs were present.

While the gene-sensing platform offers significant advancements, there are some limitations and areas for future improvement. The current system may require additional optimization in sgRNA dosage or coupling with more efficient downstream activators to improve detection limits, especially for genes with low expression levels. Lastly, future efforts should focus on refining the integration process for more complex genes and exploring ways to enable the direct activation of endogenous genes without the use of synthetic activators. These improvements could make the platform even more versatile for applications in disease-related gene sensing and therapeutic response, biomanufacturing, and real-time monitoring of gene expression.

## Data Availability

There are no new data associated with this article.
